# Neutral lipid fatty acid composition in the epidermis and blubber of long-finned pilot whales (*Globicephala melas*) in the Northeast Atlantic confirms tissue-specific stratification

**DOI:** 10.1007/s00227-026-04884-9

**Published:** 2026-06-05

**Authors:** Anna Sophie Kebke, Tessa Plint, Sascha K. Hooker, Clayton R. Magill, David J. Moore, Andrew Brownlow

**Affiliations:** 1 Scottish Marine Animal Stranding Scheme, School of Biodiversity, One Health and Veterinary Medicine, College of Medical, Veterinary and Life Sciences, University of Glasgow, Glasgow, UK; 2 Sea Mammal Research Unit, University of St Andrews, St Andrews, UK; 3 The Lyell Centre for Earth and Marine Sciences, Heriot-Watt University, Edinburgh, UK; 4 School of Physics and Astronomy, University of Edinburgh, Edinburgh, UK

**Keywords:** Biochemistry, Gas chromatography-mass spectrometry (GC-MS), Epidermis, Lipids, Cetaceans

## Abstract

Fatty acid profiling is a widely used tool in cetacean dietary and health assessments, yet most studies focus exclusively on blubber, overlooking the epidermis – the largest organ and primary barrier against environmental stressors – which remains largely uncharacterised, despite its accessibility for sampling of live and deceased animals. Here, we present a comparative analysis of fatty acid composition across the epidermis and the outer and inner blubber layer of long-finned pilot whales (*Globicephala melas*). Gas chromatography-mass spectrometry on total lipid extracts identified several major lipid classes in the cetacean epidermis: free fatty acids (~ 25%), wax and steryl esters (~ 40%), and triacylglycerols (~ 30%). Monounsaturated fatty acids (MUFAs) dominated (> 59%) across the layers. Polyunsaturated fatty acids (PUFAs), generally linked to dietary intake, were more abundant in the inner blubber layer, while saturated fatty acids (SFAs) were highest in the epidermis. Inter-layer differences in abundance of 29 out of 34 (~ 85%) identified fatty acids were statistically significant, confirming tissue-specific stratification and that epidermal and blubber fatty acid profiles are not equivalent for current fatty acid dietary analyses. The distinct fatty acid composition of the epidermis suggests roles in antimicrobial defence and thermal regulation, indicating its potential as a biomarker for monitoring the health of cryptic cetacean species under changing environmental conditions. These findings establish a baseline knowledge of the neutral lipid fatty acid profile in cetacean epidermis and reaffirm the continued importance of blubber rather than skin biopsies for dietary assessments.

## Introduction

Biochemical approaches enable us to address a wide range of questions concerning cetacean behaviour, dietary ecology, and physiological health as they provide insight into processes associated with energy acquisition and storage (Fossi and Marsili 1997; Budge et al. 2006; Atkinson et al. 2015). Energy is stored in various forms, including glucose as glycogen in liver and muscle, and lipids as triacylglycerols – of which the key constituents are fatty acids – in muscle and blubber (Budge et al. 2006; Sierra et al. 2015; Bourque et al. 2018). Mammals cannot *de novo* synthesise all fatty acids required for normal functioning of physiological processes and must instead obtain ‘essential’ fatty acids or corresponding precursors from their diet (Cook 1991). As a result, fatty acid profiles in marine mammal tissue can be used as a cost-effective tool to understand dietary patterns and trophic ecology, which can help in monitoring and understanding animals that are otherwise difficult to study (Koopman et al. 1996; Dahl et al. 2000; Hooker et al. 2001; Bories et al. 2021; Remili et al. 2022).

**Fig. 1:**
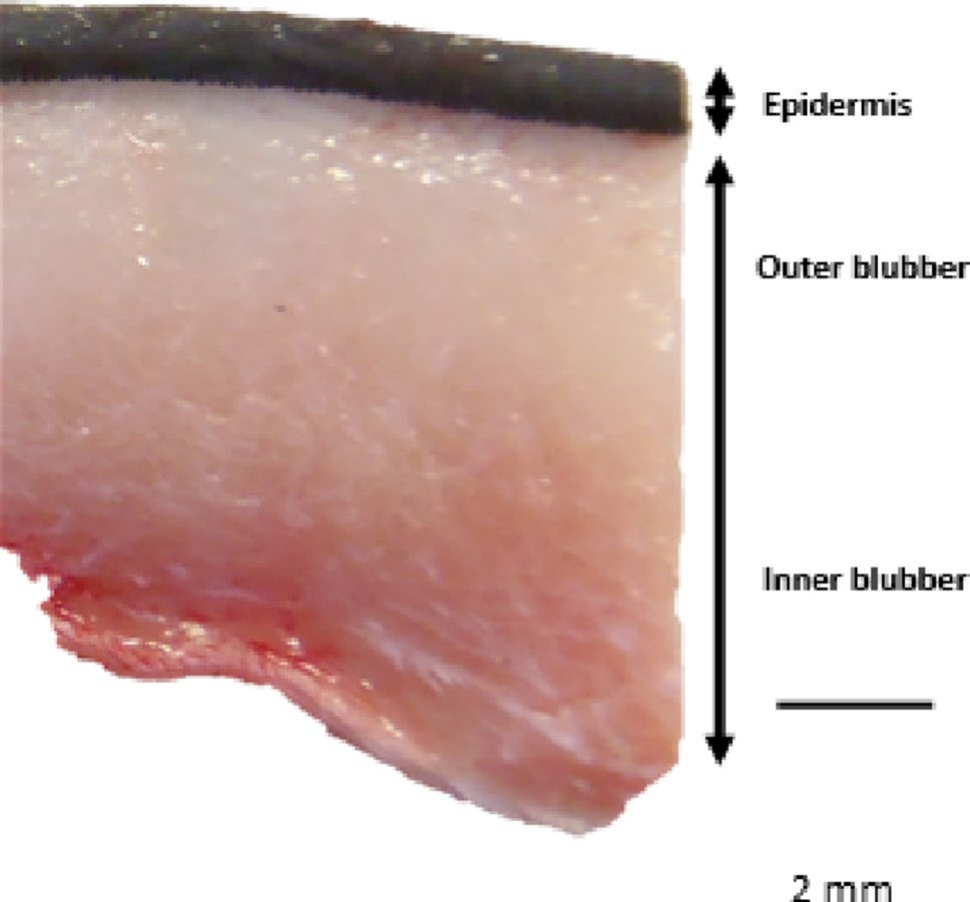
A cross-section of long-finned pilot whale (*Globicephala melas*) epidermis (skin) and blubber, identifying the representative boundaries of the outer and inner blubber layers

To date, the fatty acid composition of cetacean blubber —a specialised subcutaneous adipose tissue comprising the dermis and hypodermis — has been well characterised. Blubber exhibits unique adaptations for buoyancy, insulation, and energy storage, making it a key tissue for understanding physiological function, ecological adaptation, and health status in cetaceans (Fig. 1). Skin (also referred to as the epidermis) refers to the pigmented uppermost layer that lies directly above the dermal and hypodermal layers (the blubber) (Fig. 1) (Reeb et al. 2007). Although grossly homogeneous, blubber exhibits biochemical stratification: the outer layer, located directly beneath the epidermis, primarily functions as thermal insulation, whereas the inner layer serves as an energy reserve enabling rapid lipid mobilization (Koopman et al. 1996; Krahn et al. 2004; Samuel and Worthy 2004; Ji et al. 2019). The inner layer is more metabolically active than the outer, and the fatty acids present there are often sourced from prey and are considered the most useful for diet determination in marine mammals (Koopman et al. 1996; Iverson et al. 2004; Budge et al. 2006), but obtaining full-depth blubber samples from live cetaceans raises significant ethical, logistical, and financial considerations. Little is known about the biochemical composition of the epidermis of cetaceans, and how it compares to the blubber. There is a renewed interest in cetacean skin biochemistry from a variety of viewpoints, including epigenetic ageing techniques (Beal et al. 2019), impacts of marine pollution exposure (Lunardi et al. 2016), and the potential of using epidermis as a proxy of stress in the marine ecosystem (Fossi et al. 2014). Yet, the characterisation, composition and functions of cetacean epidermal lipids, including fatty acids, remains largely unknown. Some fatty acids in the epidermis are likely synthesised de novo by (lipo)keratinocytes (producing keratins, the proteins responsible for supporting the epidermal barrier), whilst others may be taken up through the diet and metabolised by the animal (Menon et al. 1986; Khnykin et al. 2011). As such, cetacean epidermis fatty acid composition can provide insight into not only the metabolic pathways and physiological requirements of a permanently aquatic lifestyle, but also potential insights into health status. In fact, lipidomics is a rapidly evolving research field in cetacean science and recent studies have explored the potential use of fatty acids as health markers in various cetacean species, successfully characterising fatty acids in cetacean heart and blood plasma, showing promising steps for indirect population health screening by remote sampling (Monteiro et al. 2021a). As epidermal fatty acids in humans show evidence of links between fatty acid composition and inflammatory processes (Yang et al. 2020; Knox and O’Boyle 2021), this raises questions as to whether fatty acids can also serve as proxies of cetacean health status. Here, we investigate the total lipid and fatty acid composition across the full blubber depth and epidermal profile of thirteen long-finned pilot whales (*Globicephala melas*), from which full tissue profile samples (Fig. 1) were collected from a mass stranded group (*n* = 55, 54 mortalities) on the Isle of Lewis, Scotland, in July 2023. Mass stranding events offer a rare opportunity to study gregarious cetaceans with a degree of shared life history and often an acute cause of death. These individuals stranded together, reducing potential confounding factors or biases associated with multiple single-stranded individuals, such as marked differences in recent foraging history, seasonality, stranding location and cause of death (Fernandez et al. 2012; Coombs et al. 2019; Gulland et al. 2025) as lipid content and fatty acid composition of blubber can vary with health status, environmental conditions, seasonal prey quality, and availability (Samuel and Worthy 2004; Guerrero and Rogers 2019). The animals were assessed by veterinary pathologists and considered to be in good nutritional body condition with no indication of disease contributing to their cause of death, which provides a more controlled context for initial exploratory analysis (Brownlow et al. 2026).

This study aims to characterise the epidermal lipid and fatty acid composition of a large deep-diving cetacean species. Our primary goal was to understand if the fatty acid composition of the epidermis may serve as a reliable proxy for outer and inner blubber composition. We hypothesised, based on similar studies on marine mammal blubber (Olsen and Grahl-Nielsen 2003; Strandberg et al. 2008; Skoglund et al. 2010) that fatty acid profiles would differ significantly across the epidermis, outer blubber, and inner blubber layers due to their distinct physiological roles (e.g., thermoregulation, energy reserves) and biophysical properties. However, if the epidermal fatty acid composition closely mirrors that of the inner and outer blubber layers - i.e., reliably serves as a proxy for blubber composition – this would enable broader sampling possibilities in field studies on live cetaceans, facilitating large-scale health assessments using a less invasive method for fatty acid-based ecological parameters, such as diet determination and biomarker development.

### Methodology

An opportunistic subset of thirteen individuals from the Isle of Lewis MSE were selected for this study (Table 1). Neonates and calves were not chosen for analysis to limit the confounding effects of growth and weaning on fatty acid metabolism in marine mammals (Denuncio et al. 2024). Tissue samples (< 0.5 g per individual) were taken from behind the dorsal fin. A scalpel cleaned with organic solvents was used to prevent lipid contamination from the underlying blubber layer when sampling epidermis. Subsampled tissues were held in dedicated − 20 °C freezers prior to analysis for 4–6 months and extracted by Accelerated Solvent Extractor (Dionex ASE 350). Samples were placed in stock 34 mL stainless steel cells and then extracted with 5:1 vol/vol dichloromethane: methanol (DCM: MeOH) in 3 cycles of 5 min at 100 °C, with a rinse volume of 75%. The resultant epidermal total lipid extract (TLE) was transferred to 4 mL vials, dried under gentle nitrogen stream. Subsampled TLEs were resuspended in hexane at a concentration of 10 mg/mL and were analysed for general lipid composition using ThermoScientific Trace 1300 gas chromatograph (GC) coupled to an ISQ LT single quadrupole mass spectrometer (MS; electron ionization mode; mass range of 50 to 600 amu), with helium as the carrier gas (1 mL/min) and TriPlus RSH autosampler (The Lyell Centre, Heriot-Watt University) to describe major lipid categories of the epidermis. Individual lipids were identified using standard reference materials (e.g., Schimmelmann A5, F8-3), when available, or otherwise via a combination of electron impact (EI) mass spectra and Dool-Kratz retention index values (webbook.nist.gov).

**Table 1:** Associated data (sample ID, sex, body length, and age class as determined by length) for long-finned pilot whale (*Globicephala melas)* (*n = 13*) included in this study

Sample ID	Sex	Body length (cm)	Age class
M371.10/23	Male	602	Adult
M371.13/23	Female	430	Adult
M371.15/23	Female	472	Adult
M371.25/23	Female	447	Adult
M371.27/23	Male	444	Adult
M371.28/23	Male	510	Adult
M371.30/23	Female	400	Adult
M371.31/23	Male	380	Juvenile
M371.35/23	Female	360	Adult
M371.40/23	Female	450	Adult
M371.44/23	Male	529	Adult
M371.47/23	Male	406	Adult
M371.50/23	Female	425	Adult

Fatty acids from each tissue layer were isolated and derivatised for identification and (semi)quantification. An aliquot of TLE from each tissue sample was subjected to acid methanolysis to cleave intact neutral lipids, and transesterify them into component fatty acid methyl esters (FAMEs). Hydrolysis was achieved through lipid reaction with 250 uL of 0.5 N methanolic hydrochloric acid at 60 °C for 16 h. The hydrolysis reaction was quenched with 250 uL of DCM-extracted ultra-clean Millipore water. The subsequent liquid-liquid extraction of the organic material was performed using four rinses of 500 uL of 4:1 vol/vol hexane: DCM. The resultant FAMEs were dried under gentle nitrogen stream and resuspended in 1.5 mL of hexane.

For all tissues, fatty acid occurrence, identification, and distribution (viz. abundance) were analysed using splitless injection (1 µL) onto a ThermoScientific Trace 1300 gas chromatograph (GC) coupled to an ISQ LT single quadrupole mass spectrometer (MS; electron ionization mode; mass range of 50 to 600 amu), with helium as the carrier gas (1 mL/min) and TriPlus RSH autosampler (The Lyell Centre, Heriot-Watt University). The GC was equipped with a 30 m DB-5SilMS column (0.25 μm film × 0.25 mm internal diameter). GC oven temperature was ramped from 60 °C at 6 °C/min to 320 °C and held for 10 min at final temperature.

Individual FAMEs were identified using internationally accepted reference materials (Supelco 37 Component FAME Mix [CRM47885] and Schimmelmann FAME Mixture F8-3), comparison with electron ionisation mass spectra (NIST 2017 library), and relative retention index and theoretical elution order (Eder 1995; Härtig 2008). Identifications were confirmed by comparison with parent ions and fragmentation patterns available on the Lipid Web (www.lipidmaps.org). The processing software was ThermoScientific Chromeleon Chromatography Data System (CDS) version 7.3.1. Fatty acids are reported as relative abundance (%) of total identified fatty acid compounds, and mean values summarised for each fatty acid per tissue type (epidermis, outer, and inner blubber).

Fatty acids are referred to using standard nomenclature of their carbon chain length followed by number of double bonds and, if necessary, position of bonds or associated functionalisation. For instance, the 18-carbon long fatty acids with double bonds at positions 9, 12, and 15 –commonly known as α-linolenic acid and more formally called 9,12,15-octadecatrienoic acid – would be denoted as C18:3(*n*−3) here. All double-bonds are assumed to be *cis* (as opposed to *trans*) isomers unless otherwise noted as cis stereoisomerism dominates undegraded biological lipids (Chatgilialoglu et al. 2014). Additionally, SFA is used to indicate saturated fatty acids (fatty acids with no double bonds), MUFA (monounsaturated fatty acids) indicates fatty acids with one double bond, and PUFA compounds (polyunsaturated fatty acids) is used to describe fatty acids with ≥ 2 double bonds.

Fatty acid proportions were calculated individually for each tissue, alongside the total amount of saturated (SFA), monounsaturated (MUFA) and polyunsaturated (PUFA), and proportions (in percentage of total identified fatty acid methyl esters, FAMEs) and their corresponding summary variables (total SFA, MUFA, PUFA) summarised. The *jitter_geom* function in R package *ggplot2* (version 3.5.0) (Wickham 2016) was used on log-transformed abundance data to add a small amount of random variation to each point to avoid over-plotting.

The non-normal nature of the data required a Friedman test, a non-parametric method that ranks data within individuals and does not assume normality, to assess systematic differences in fatty acid abundance across tissue layers (inner blubber, outer blubber, and epidermis) (Hoffman 2015). This approach is suitable for repeated-measures data involving more than two related groups (three tissue types in this study) and returns a chi-squared statistic (χ²). All statistical analysis was performed in Rstudio (v 4.3.3) (R Core Team 2025).

## Results

**Fig. 2:**
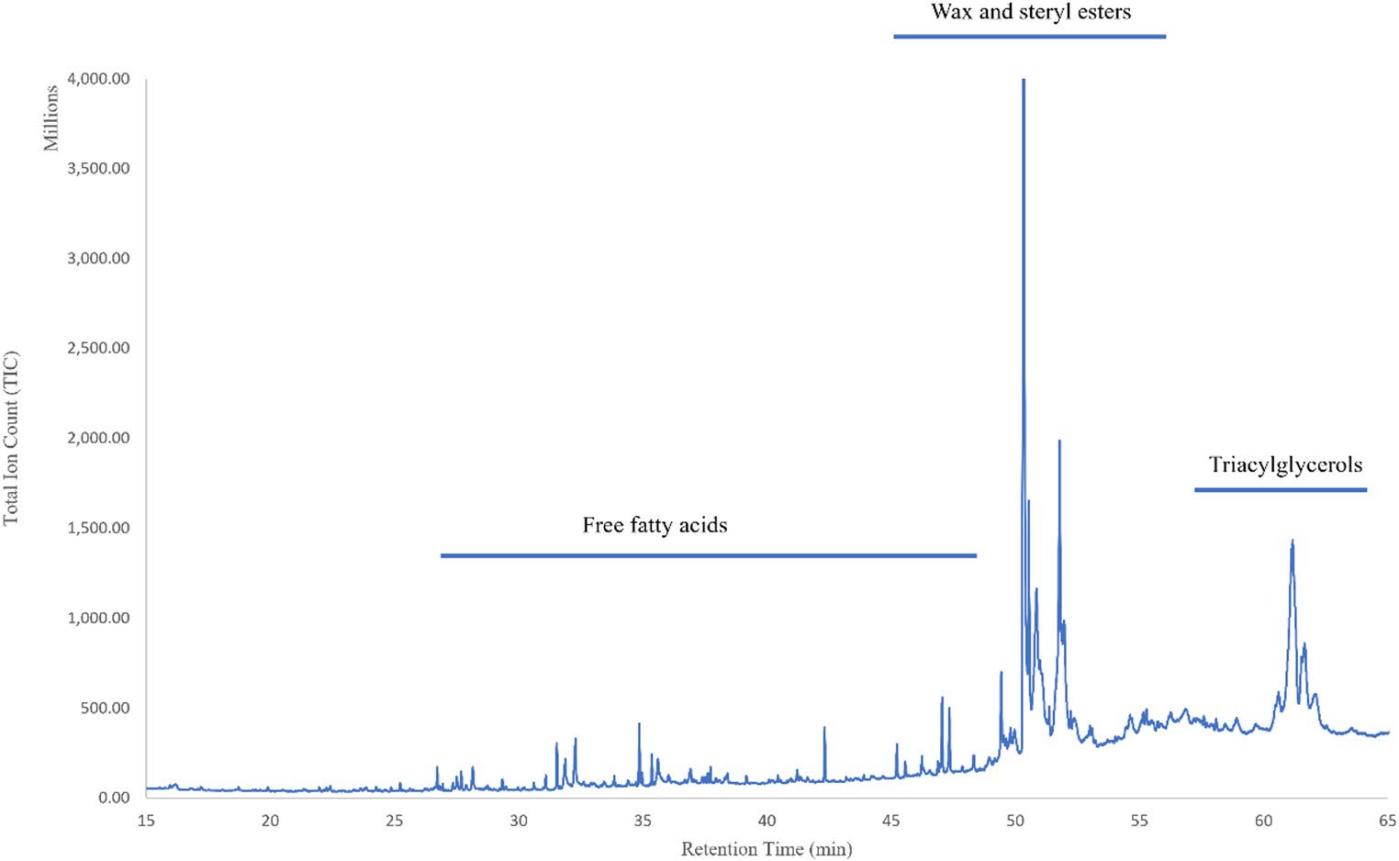
Representative gas chromatography-mass spectrometry (GC-MS) chromatogram total lipid extract from long-finned pilot whale (*Globicephala melas*) epidermis, depicting three major lipid classes: free fatty acids, wax and steryl esters, and triacylglycerols

**Fig. 3:**
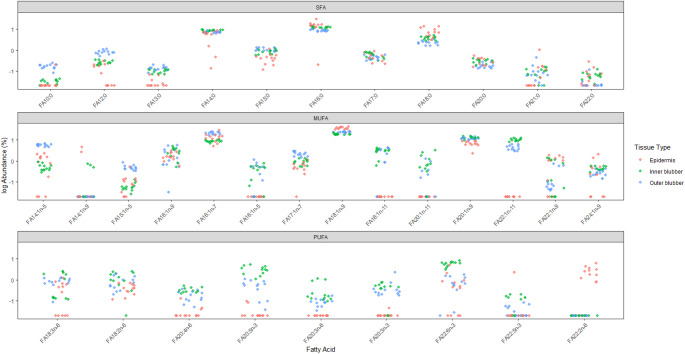
Fatty acid abundance (log transformed and categorized by fatty acid class (saturated fatty acids; SFAs, monounsaturated fatty acids; MUFAs, polyunsaturated fatty acids; PUFAs, %) in inner blubber (red), outer blubber (green) and epidermis (blue) in long-finned pilot whales (*Globicephalas melas*), northeast Atlantic (*n* = 13)

The representative average composition of total lipid extract (TLE) components in the epidermis showed that free fatty acids comprised approximately 25% of total lipids, while wax and steryl esters together accounted for about 40% (Fig. 2). Triacylglycerols (TAG) account for the remaining lipid content (~ 30%) in epidermal samples, although this value is likely an underestimate of true abundance as a myriad of TAGs have boiling points well above the maximum temperature threshold of our GC column (320 °C), and as a result, it was not possible to quantify these remaining TAGs. The outstanding 5% lipid components include alkyl lipids such as alkanes and alcohols. Our results demonstrate that the epidermal fatty acid profile differed significantly from those of both the inner and outer blubber layers, while still exhibiting broadly similar patterns across fatty acid classes: monounsaturated fatty acids (MUFAs) were most abundant across all three tissue layers (> 59% in all layers), followed by saturated fatty acids (SFAs, 20–30%) and polyunsaturated fatty acids (PUFAs, 3–12%), respectively (Table 2). The percent relative abundance contributions of SFAs were higher in the epidermis than the outer and inner blubber (29.8%) (Table 2). In contrast, the total contribution of PUFAs was higher in inner blubber (12.3%) compared to the epidermis and outer blubber (3.4% and 3.3%, respectively) (Table 2). A total of 34 fatty acids were identified in the blubber layers with percentage contributions greater than 0.5% (Table 3). The Friedman test revealed significant differences (*p* < 0.05) in fatty acid abundance across tissues for 29 out of 34 (~ 85%) of the fatty acids (Table 3). The relative abundance of PUFAs decreased in a gradient-like pattern from the inner blubber to the epidermis (Fig. 3; Table 3). The test statistic (χ²) reflects the degree of consistency in rank differences across individuals, with higher values indicating stronger evidence of tissue-specific variation. The most abundant fatty acid across tissue layers was fatty acid 18:1n-9 (Fig. 3; Table 3). In contrast, fatty acid 22:2n-6 was the least abundant and only detected in the epidermis, with trace amounts in the outer and inner blubber layers.

**Table 2:** The relative abundance (%) of different fatty acid classes (saturated fatty acids; SFAs, monounsaturated fatty acids; MUFAs, polyunsaturated fatty acids; PUFAs) across tissue layers (inner blubber, outer blubber, and epidermis) in long-finned pilot whales (*Globicephala melas*), northeast Atlantic (*n* = 13)

Tissue	SFAs Relative abundance (%)	MUFAs Relative abundance (%)	PUFAs Relative abundance (%)
Inner blubber	27.39	59.68	12.33
Outer blubber	20.89	74.33	3.29
Epidermis	29.84	60.88	3.42

**Table 3:** Results of Friedman tests comparing fatty acid content (% relative abundance) across inner blubber, outer blubber and epidermis from long-finned pilot whales (*Globicephala melas*) in the Northeast Atlantic (*n =* 13)

Fatty acid	Mean abundance in inner blubber (%) ± SD	Mean abundance in outer blubber (%) ± SD	Mean abundance in epidermis (%) ± SD	*p*-value	χ² (df = 2)
**SFAs**					
FA10:0	0.03 ± 0.01	0.17 ± 0.04	Trace	**< 0.001**	17.9
FA12:0	0.25 ± 0.05	0.78 ± 0.18	0.12 ± 0.11	**< 0.001**	20.7
FA13:0	0.11 ± 0.02	0.14 ± 0.03	Trace	**< 0.001**	20.7
FA14:0	8.91 ± 0.80	7.27 ± 0.79	6.38 ± 2.98	**< 0.001**	15.5
FA15:0	0.86 ± 0.21	1.02 ± 0.19	0.41 ± 0.26	**< 0.001**	22.2
FA16:0	12.12 ± 1.15	8.50 ± 0.82	15.37 ± 6.78	**< 0.001**	18.5
FA17:0	0.64 ± 0.19	0.46 ± 0.11	0.41 ± 0.20	**0.017**	8.7
FA18:0	4.0 ± 0.70	2.32 ± 0.46	6.73 ± 3.73	**< 0.001**	20.7
FA20:0	0.32 ± 0.06	0.17 ± 0.05	0.33 ± 0.12	**0.001**	15.2
FA21:0	0.08 ± 0.05	0.06 ± 0.12	0.02 ± 0.29	0.64	0.89
FA22:0	0.06 ± 0.02	Trace	Trace	**0.005**	10.5
FA24:0	0.01 ± 0.01	Trace	Trace	**0.021**	7.71
**MUFAs**					
FA14:1n-5	0.45 ± 0.20	5.96 ± 0.72	1.14 ± 0.84	**< 0.001**	18.2
FA15:1n-5	0.05 ± 0.03	0.44 ± 0.15	0.09 ± 0.14	**< 0.001**	18.2
FA16:1n-9	2.59 ± 1.50	1.21 ± 1.95	1.59 ± 0.78	0.264	2.67
FA16:1n-7	8.89 ± 0.90	21.55 ± 2.72	13.08 ± 6.33	**< 0.001**	17.2
FA16:1n-5	0.47 ± 0.25	0.38 ± 0.40	Trace	**0.001**	13.8
FA17:1n-7	0.92 ± 0.28	2.22 ± 0.50	0.55 ± 0.33	**< 0.001**	19.5
FA18:1n-9	18.94 ± 1.40	23.74 ± 1.17	36.31 ± 5.07	**< 0.001**	22.2
FA18:1n-11	3.17 ± 1.08	2.74 ± 1.75	Trace	**0.001**	14.8
FA20:1n-11	0.64 ± 1.04	0.08 ± 0.42	Trace	**< 0.001**	16.2
FA20:1n-9	12.82 ± 1.60	11.58 ± 1.34	6.83 ± 1.89	**< 0.001**	19.5
FA22:1n-11	10.22 ± 1.47	4.10 ± 1.18	Trace	**< 0.001**	24
FA22:1n-9	0.51 ± 0.60	0.20 ± 0.26	0.91 ± 0.70	0.144	3.87
FA24:1n-9	0.41 ± 0.13	0.26 ± 0.09	0.46 ± 0.66	0.205	3.17
**PUFAs**					
FA18:3n-6	0.62 ± 1.04	0.88 ± 0.34	0.36 ± 0.40	**0.017**	8.17
FA18:2n-6	1.18 ± 0.84	0.63 ± 0.58	0.40 ± 0.20	**0.013**	8.67
FA20:5n-3	3.08 ± 1.31	0.57 ± 0.34	Trace	**< 0.001**	22.2
FA20:4n-6	0.29 ± 0.09	0.16 ± 0.11	Trace	**< 0.001**	19.5
FA20:3n-6	0.16 ± 0.40	0.09 ± 0.04	Trace	**< 0.001**	22.2
FA20:3n-3	0.46 ± 0.27	0.27 ± 0.60	Trace	**0.001**	13
FA22:6n-3	6.41 ± 1.49	0.68 ± 0.58	0.47 ± 1.43	**< 0.001**	19.5
FA22:5n-3	0.13 ± 0.08	0.01 ± 0.03	Trace	0.053	5.88
FA22:2n-6	Trace	Trace	2.19 ± 1.76	**< 0.001**	22

The Friedman test statistic (χ²) and associated degrees of freedom (*df* = 2) are reported for each identified fatty acid. Significant results (*p* < 0.05) are shown in bold

## Discussion

Total lipid extract (TLE) components in long-finned pilot whale epidermis indicate that wax and steryl esters accounted for almost 40% of total lipids, followed by triacylglycerols (~ 30%) and free fatty acids (~ 25%) (). This is in stark contrast to the lipid composition reported in long-finned pilot whale blubber, where triacylglycerols account for ~ 95% of total lipids (Koopman 2018), and results from other cetacean species which also show high levels of triacylglycerols in blubber (Krahn et al. 2004; Bories et al. 2021). The levels of free fatty acids in the epidermis are relatively high compared to terrestrial mammalian epidermis. This may be explained by the free fatty acids being accumulated in the cells of the thick epidermis instead of being transported into the intercellular spaces, or, alternatively, this could be a result of free fatty acid accumulation consequent to enzymatic decomposition during sample storage (Menon et al. 1986; Elias et al. 1987; Pfeiffer and Jones 1993; Meyer 2010, 2012).

The epidermal fatty acid profile in long-finned pilot whales is chemically distinct, showing significant stratification (29 out of 34 fatty acids) relative to both inner and outer blubber layers. This confirms that epidermal fatty acid profiles are not equivalent to either the inner or outer blubber layers, and as such reaffirms that the quantification and characterisation of epidermal fatty acids cannot be used as a proxy for dietary fatty acids instead of blubber.

However, the identification of fatty acids in long-finned pilot whale skin reported here may be useful for compound-specific stable isotope analysis (CSIA) of fatty acids. CSIA builds upon the isotope values of individual monomers that constitute a macromolecule (e.g., fatty acids in lipids, or amino acids in protein), and can provide a high level of dietary information (Nielsen et al. 2018). In contrast to bulk stable isotope analysis, CSIA allows for the separation of effects from baseline isotopic variation to those related to trophic processes. Whilst relatively well established for dietary determination in several cetacean species (e.g., Pomerleau et al. 2017; Troina et al. 2021), there is a significant knowledge gap for continued CSIA on individual fatty acids due to the logistical and financial constraints on obtaining fatty acid compositions and characterising these in an animal. We hope that, by reporting individual fatty acids in the tissues of long-finned pilot whales, our study has helped facilitate future work using CSIA on fatty acids.

All but one of the polyunsaturated fatty acids (PUFAs) (indicative of diet) were significantly different between tissues. Generally, PUFAs were most abundant in the inner blubber layers, highlighting the reported selective deposition of dietary-derived lipids within this more metabolically active tissue. Monounsaturated fatty acids (MUFAs) were most prevalent in the outer blubber layers and epidermis – suggesting a high accumulation of endogenously synthesised fatty acids in these tissues. Notably, fatty acid 18:1n-9 (a MUFA) had the highest relative abundance across all three tissues. This result was expected due to the prevalent role of 18:1n-9 (also known as oleic acid) in nature due to its central role in metabolism, energy storage and function in cell membranes (Gurr et al. 2016; Smith 2024). Long-finned pilot whale blubber from other studies (Walters 2005; Monteiro et al. 2015; Joensen 2022) also showed high abundances of this fatty acid.

The gradient-like pattern of fatty acids across the blubber and epidermal layers is congruent with that of other marine mammals (e.g., pinnipeds, cf., Käkelä and Hyvärinen 1993; Olsen and Grahl-Nielsen 2003; Strandberg et al. 2008; Skoglund et al. 2010; Bourque et al. 2018). In cetaceans, Madin et al. 2022 found a similar stratification pattern when comparing blubber and epidermal fatty acids of the southern fin whale (*Balaenoptera physalus quoyi*). However, they did not find any significant differences in the abundance of fatty acids between the epidermis and blubber, but only analysed one individual and did not differentiate between inner and outer blubber layers. In addition, the animal analysed by Madin et al. (2022) was living in warmer waters (the North Pacific Ocean) than the animals analysed here. This meant that differences in water temperatures could have influenced and modified fatty acid composition and distribution patterns across tissues as a thermoregulatory response, as a higher level of fatty acid desaturation is expected with colder water temperatures and not necessarily related to diet alone (Sakamoto and Murata 2002; Guerrero and Rogers 2019).

The epidermis and blubber layers in vivo clearly do not operate in isolation but as components of an integrated biological system; consequently, the interpretation of their distinct biochemical characteristics—particularly fatty acid composition—requires an integrated metabolic model that accounts for their functional interdependence within the animal. Marine mammals need to combat heat loss in cold aquatic environments, and it is possible that the fatty acids reported here may be stratified across the tissues according to their melting points. In long-finned pilot whales, repeated foraging dives expose the animals to substantial temperature gradients from surface waters to ocean depths, further compounded by the increased pressure at depth. This would have extreme impacts on the epidermis, and biochemical mechanisms are likely to have a critical role in maintaining thermal homeostasis and functional integrity in a permanently aquatic environment. Chemically, unsaturated fatty acids (MUFAs and PUFAs) have lower melting points than saturated fatty acids (SFAs) of the same chain length, with the melting point increasing alongside the chain length. As the fluidity of membranes is directly correlated with the amount of unsaturated fatty acids in the membrane (Irving and Hart 1957; Sakamoto and Murata 2002; Guschina and Harwood 2009; Guerrero and Rogers 2019), the tissues may maintain their fluidity for longer in colder temperatures and by extension preventing rigidity thus protecting the warmer inner layers. The fatty acid stratification pattern observed across the integument may therefore reflect a physiological need to maintain tissue fluidity and function in a cold, permanently aquatic environment through various tissue-lipid responses mediated by fatty acid synthesis. The abundance of MUFAs in blubber may vary in response to environmental temperatures: for example, short-chained MUFAs have low melting points and serve as good insulators from the surrounding cold environment, explaining their relatively high abundance in both the epidermal and outer blubber layers (Guerrero and Rogers 2019; Tang et al. 2022). PUFAs in epidermal lipids contribute to the fluidity of membranes and are essential for the growth of cells at low temperatures (Sakamoto and Murata 2002), but are difficult for mammals to synthesise and route due to their complex structures. In turn, this complex process would create a thermal gradient preventing heat loss, and as such, it might be important for the animal to maintain high levels of MUFAs in the outer blubber and epidermis in a cold environment, explaining the pattern reported here (a high median abundance of MUFAs across all tissues), but as ocean temperatures rise due to climate variability and climate change, this pattern of fatty acid stratification might over time shift in response to warming waters.

Whilst largely exploratory, this study also raises the possibility of using epidermal fatty acids as a biomarker for health. The evidence in humans suggests a link between epidermal fatty acid compositions and inflammatory processes (Yang et al. 2020; Knox and O’Boyle 2021). The outermost epidermis is generally an inhospitable environment for microbes due to its mild acidity (the so-called “acid mantle”), with one of its main antimicrobial properties residing in the lipid fraction of the epidermis, likely as free fatty acids (Drake et al. 2008; de Szalay and Wertz 2023). Certain fatty acids, particularly SFAs and MUFAs (e.g., C16:0, C16:1n-7, and C18:1) have demonstrated antimicrobial properties against various pathogenic bacteria and fungal pathogens in a laboratory setting (Drake et al. 2008; Fischer et al. 2014; Prawira et al. 2022). From a health perspective, deficiencies in epidermal fatty acids in humans, especially MUFAs, have been linked to increased susceptibility to pathogenic colonisation, highlighting the importance of a functioning lipid barrier as an epidermal defence mechanism (Wille and Kydonieus 2003; Takigawa et al. 2005; Becam et al. 2017). In humans, these fatty acids are derived from sebaceous glands. However, these structures are absent in cetaceans, raising further questions about fatty acid synthesis and dynamics in cetacean epidermis (Feingold 2009; Springer et al. 2021). Nonetheless, it is likely that some epidermal free fatty acids (alongside the high epidermal surface sloughing properties, contributing to a quick cetacean epidermis turnover rate) can help protect cetaceans against the settlement of external organisms, that can disrupt the epidermal integrity (i.e., microbes or barnacle larvae; Baum et al. 2002, 2003; Hachem et al. 2003). Environmental stressors might also influence epidermal lipid composition, as the pressures of increasing sea surface temperatures and chemical pollution have already been established in cetaceans, with data from UK-stranded short-beaked common dolphins (*Delphinus delphis)* showing PCB concentrations and sea surface temperatures associated with an increased risk of infectious disease mortality (Williams et al. 2025). This highlights the relevance of this exploratory research, as both ocean warming and chemical pollution can disrupt the epidermal lipid barrier, making the animal more susceptible to external pathogens (Haubold et al. 2000; Van Bressem et al. 2009; Mouton et al. 2012, 2015). This further emphasises the necessity of epidermal defence in a changing environment, and the importance of understanding the underlying fatty acid dynamics of the epidermis. It is important, however, to note that we have only reported neutral lipids (fatty acids) here due to the system and methodologies used (GC-MS and the transesterification of lipid extracts to create fatty acid methyl esters). As a result, we have not reported the full lipid profile, as polar lipids such as phospholipids and ceramides have not been characterised. Whilst we recognise that the resultant fatty acid profile would reflect composite contributions from all lipid classes, we do not know how prominent polar lipids are in the epidermis. As such, the fatty acids derived from polar lipid classes might not make a sufficient contribution to total lipids to be useful as potential health biomarkers. Future studies wishing to investigate compositional distribution profiles in-depth might find it helpful to separate component fatty acids of different lipid classes via gravity-column chromatographic isolation, or through liquid-chromatography-mass spectrometry, which is better suited for polar compounds in complex biological samples.

Due to the small sample size and non-uniform distribution of sexes and age classes within our exploratory sample set, we could not investigate the different effects of sex and age on fatty acid composition across the three tissues. However, this would be an interesting addition to future studies wishing to investigate fatty acid dynamics in cetacean tissue. It would also be interesting to investigate whether epidermal microbiome and fatty acid composition vary seasonally, as studies on other cetaceans suggest microbiome changes with diet and season (Apprill et al. 2014; Bierlich et al. 2018). Additionally, identifying the epidermal fatty acid composition of individuals with known skin disease would expand on current knowledge of lipid barrier function in cetaceans, and provide insight on disruption to the protective barrier function of the epidermis. By characterising the epidermal, inner, and outer blubber fatty acids in long-finned pilot whales, this study expands our understanding of the role of lipid biochemistry in cetacean physiology. The observed stratification likely reflects a complex interplay of dietary input, endogenous synthesis, and environmental adaptations. We identified that the epidermis fatty acids are not an equivalent proxy for the inner and outer blubber layers. As such, epidermal fatty acids are not a suitable alternative for dietary studies reliant on inner and outer blubber fatty acid data but may provide valuable insights into the roles of lipids and fatty acids in the cetacean epidermal barrier, and we hope that our results may lend themselves to future applications of compound-specific stable isotope analysis on fatty acids. In addition, we quantify the composition of healthy epidermal fatty acids, and highlight the potential of the epidermis as a candidate for future biomarker development as technological innovations advance.

## Data Availability

All necessary data is included in the article. Further inquiries can be directed to the corresponding author.
